# Dorsal vs. ventral differences in fast Up-state-associated oscillations in the medial prefrontal cortex of the urethane-anesthetized rat

**DOI:** 10.1152/jn.00762.2016

**Published:** 2016-12-21

**Authors:** Sabine Gretenkord, Adrian Rees, Miles A. Whittington, Sarah E. Gartside, Fiona E. N. LeBeau

**Affiliations:** ^1^Institute of Neuroscience, Newcastle University, Medical School, Newcastle-upon-Tyne, United Kingdom;; ^2^Developmental Neurophysiology, Institute of Neuroanatomy, University Medical Center Hamburg-Eppendorf, Hamburg, Germany; and; ^3^York-Hull Medical School, F1-Department of Biology, York University, Heslington, United Kingdom

**Keywords:** prefrontal cortex, Up-state, slow oscillations, spindles, gamma oscillations, rat

## Abstract

Cortical slow oscillations (0.1–1 Hz), which may play a role in memory consolidation, are a hallmark of non-rapid eye movement (NREM) sleep and also occur under anesthesia. During slow oscillations the neuronal network generates faster oscillations on the active Up-states and these nested oscillations are particularly prominent in the PFC. In rodents the medial prefrontal cortex (mPFC) consists of several subregions: anterior cingulate cortex (ACC), prelimbic (PrL), infralimbic (IL), and dorsal peduncular cortices (DP). Although each region has a distinct anatomy and function, it is not known whether slow or fast network oscillations differ between subregions in vivo. We have simultaneously recorded slow and fast network oscillations in all four subregions of the rodent mPFC under urethane anesthesia. Slow oscillations were synchronous between the mPFC subregions, and across the hemispheres, with no consistent amplitude difference between subregions. Delta (2–4 Hz) activity showed only small differences between subregions. However, oscillations in the spindle (6–15 Hz)-, beta (20–30 Hz), gamma (30–80 Hz)-, and high-gamma (80–150 Hz)-frequency bands were consistently larger in the dorsal regions (ACC and PrL) compared with ventral regions (IL and DP). In dorsal regions the peak power of spindle, beta, and gamma activity occurred early after onset of the Up-state. In the ventral regions, especially the DP, the oscillatory power in the spindle-, beta-, and gamma-frequency ranges peaked later in the Up-state. These results suggest variations in fast network oscillations within the mPFC that may reflect the different functions and connectivity of these subregions.

**NEW & NOTEWORTHY** We demonstrate, in the urethane-anesthetized rat, that within the medial prefrontal cortex (mPFC) there are clear subregional differences in the fast network oscillations associated with the slow oscillation Up-state. These differences, particularly between the dorsal and ventral subregions of the mPFC, may reflect the different functions and connectivity of these subregions.

## Abstract (precis)

We demonstrate, in the urethane-anesthetized rat, that within the medial prefrontal cortex (mPFC) there are clear subregional differences in the fast network oscillations associated with the slow oscillation Up-state. These differences, particularly between the dorsal and ventral subregions of the mPFC, may reflect the different functions and connectivity of these subregions.

cortical slow oscillations, characterized by transitions between Up- and Down-states (UDS) evident in the local field potential (LFP) or electroencephalogram (EEG), are seen during natural sleep, as well as under ketamine and urethane anesthesia (Steriade et al. 1993; Destexhe et al. 1999; Chauvette et al. 2010; Barthó et al. 2014). During UDS the membrane potential of cortical pyramidal cells is depolarized and hyperpolarized, respectively, with firing occurring predominantly on the depolarized Up-state (Steriade et al. 1996a,b; Sanchez-Vives and McCormick 2000; Hughes et al. 2002). Slow oscillations are synchronous over wide cortical areas (Isomura et al. 2006; Volgushev et al. 2006; Ruiz-Mejias et al. 2011) and are thought to play a role in the transfer of information, and memory consolidation (Marshall et al. 2006; Stickgold 2006; Peyrache et al. 2009; Diekelmann and Born 2010; Johnson et al. 2010). Faster oscillatory activities, including spindle (6–15 Hz)-, beta (15–30 Hz)-, gamma (30–80 Hz)-, and high-gamma (80–150 Hz)-frequency activity, are amplitude-phase coupled with the Up-state (Compte et al. 2008; Hasenstaub et al. 2005; Ruiz-Mejias et al. 2011; Valencia et al. 2013), but their precise roles when nested within slow oscillations are still unclear.

In rodents, the dorsal anterior cingulate cortex (ACC) and prelimbic (PrL) and ventral infralimbic (IL) and dorsal peduncular cortex (DP) regions of medial prefrontal cortex (mPFC) (Paxinos and Watson 2007) are distinguished by their distinct cytoarchitectures (Gabbott et al. 2005; Vogt and Paxinos 2014). In addition, each subregion has different inputs and outputs (Gabbott et al. 2007; Hoover and Vertes 2007; Vertes et al. 2015; Kuramoto et al. 2016) and has different functional roles (Heidbreder and Groenewegen 2003; Kesner and Churchwell 2011; Vertes et al. 2015). In particular, ACC and PrL play a role in attention and working memory, while the more ventral IL has been implicated in goal-directed behavior and autonomic functions (Miller and Cohen 2001; Heidbreder and Groenewegen 2003; Seamans et al. 2008; Kesner and Churchwell 2011; Euston et al. 2012; Parnaudeau et al. 2013). The function of the DP currently remains unclear.

Previous work, in the anesthetized mouse, has shown that slow and fast oscillations generated in the mPFC differ from those in sensory and motor cortical areas in several respects (Ruiz-Mejias et al. 2011). These include greater power in the beta- and gamma- frequency range, higher presumed pyramidal cell firing rates during the Up-state, and a faster transition to UDS (Ruiz-Mejias et al. 2011). Although several in vitro studies have highlighted subregional differences within mPFC in fast network activity (van Aerde et al. 2008; Glykos et al. 2015), to date no studies have compared slow oscillations and nested fast network activity within the mPFC subregions in vivo either in natual sleep or under anesthesia. We predicted that the distinct functions and connectivity of mPFC subregions might be reflected in subregional differences in slow and/or fast oscillations. To test this hypothesis we recorded network activity from layer III in all subregions of the mPFC simultaneously under urethane anesthesia and compared the slow oscillations and the fast oscillations associated with the Up-states within the mPFC. Our findings revealed significant differences between mPFC subregions, particularly when comparing the dorsal ACC and PrL regions vs. the most ventral region, DP.

## METHODS

### 

#### Animals.

All procedures described below were independently reviewed in accordance with the UK Animals (Scientific Procedures) Act 1986 and the European Union Directive 2010/63/EU. Male Hooded Lister rats were supplied by Charles River Laboratories (Margate, Kent, UK) and housed at Newcastle University’s animal facility in a temperature- and humidity-controlled environment consistent with the ARRIVE (Animal Research: Reporting of In Vivo Experiments) guidelines. Rats were kept in an enriched environment (cage toys) under a 12-h light-dark cycle (lights on 7 AM-7 PM) with access to food and water ad libitum. Rats were housed in cages of two to four and were allowed a week of acclimatization before the experiment. Experiments were commenced ~2 h into the light (sleep) phase of the circadian cycle.

#### Anesthesia and surgery.

Rats weighing 250–330 g were anesthetized with urethane (Sigma-Aldrich. St. Louis, MO). An initial dose of 1.5–1.9 g/kg was administered by intraperitoneal injection. Additional doses of 0.5 g/kg ip were given every half an hour until a deep anesthesia level (confirmed by absence of the pedal withdrawal reflex) was achieved.

After a sufficient depth of anesthesia was achieved, the animal was fixed in a stereotaxic frame (Kopf, Tujunga, CA). A heating pad with feedback temperature control via a rectal probe (Harvard Apparatus, Holliston, MA) maintained the core temperature of the rat at 36.8°C. A pulse oximeter was attached to the animal’s hind paw and blood oxygen saturation was monitored (Physiosuite, Kent Scientific, Torrington, CT). The animal breathed spontaneously, but to maintain an oxygen saturation of >90%, medical oxygen (BOC Industrial Gases, UK) was supplied through a tube mounted to the nose bar of the stereotaxic frame. A skin incision was made in the scalp and infused with lidocaine before the fibrous tissue and periosteum were retracted to expose bregma. A craniotomy was drilled above the mPFC of both hemispheres. Dual shank (1,000-μm separation) 16-channel silicon probes (8 recording sites per shank, 500-μm inter-site spacing) were used for recording (E16-500-S02-1000-L7.5; Atlas Neuroengineering, Leuven, Belgium). Before insertion, the silicon probes were coated with the fluorescent dye DiI (1,1′-dioctadecyl-3,3,3′,3′-tetramethylindocarbocyanine; Molecular Probes, Eugene, OR), dissolved in DMSO (1.5–2.5 mg/ml). The probes were implanted into the left and right mPFC **(**AP: +2.3–2.5, ML: ±0.5, DV: 5.3 mm) through the dura, taking particular care not to damage the superior sagittal sinus that lies in the midline. The electrode was lowered in the *z*-plane using a one-axis oil-filled hydraulic micromanipulator (Narishige, East Meadow, NY).

#### Data recording and acquisition.

Field potential recordings were made from two sites in each subregion of the mPFC: ACC, PrL, IL, and DP in both hemispheres. As each region contained two recording sites these are distinguished throughout as dorsal (d) or ventral (v) for each subregion. Post hoc histology confirmed that the shanks were equidistant from the midline so that the laminar position of the shank was in layer III of the mPFC in both hemispheres. Each of the 16 channels of extracellular signal passed through a unity-gain headstage (Plexon, Dallas, TX) and was then amplified (×1,000) and filtered (0.07–300 Hz for LFP) by a Plexon preamplifier. The LFP was digitized at 1,000 Hz and recorded on a PC (DELL Intel 4-core) running Plexon software (Sort Client).

#### Histological verification of recording site position.

After the experiment the rat was killed by injection with Euthatal (200 mg/ml ip). The brain was removed from the skull and post fixed in 4% paraformaldehyde (PFA), 0.1 M phosphate-buffered solution (PBS) at 4°C for a minimum of 12 h before cryoprotection in 30% sucrose solution for a maximum of 24 h until it sank. Coronal sections (60–100 μm) were cut on a cooled vibratome (Zeiss Hyrax V50; Zeiss, Oberkochen, Germany) and collected in 0.1 M PBS. To visualize the cytoarchitecture, freely floating sections were then stained using either green fluorescent Nissl stain (NeuroTrace 500/525; Molecular Probes) or bisbenzimide H33258 (Sigma-Aldrich). After being stained, sections were mounted and coverslipped using Vectashield HardSet mounting medium (Vector Laboratories, Peterborough, UK).

Photographs were taken using an Axio Imager Z2 microscope (Zeiss) and Axiovision 4.8 software (Zeiss). Tiled pictures were taken using the mosaic setting, with ×2.5 magnification. The rhodamine filter was used to reveal the DiI staining of the electrode tract. The fluorescein isothiocyanate (FITC) filter was used for the green fluorescent Nissl and the 4′,6-diamidine-2-phenylindol (DAPI) filter for the bisbenzimide stain. An overlay image was produced using Axiovison software.

#### Data preprocessing.

The raw LFP signal contained 50-Hz mains noise as well as higher frequency harmonics and was therefore notch filtered on all channels between 48 and 52, 98 and 102, and 148 and 152 Hz using a linear-phase finite impulse response filter (FIR) created using “fdatool.m” in combination with the MATLAB (Mathworks, Nantick, MA) “filtfilt.m” function. The filtering resulted in removal of a 4-Hz band from the gamma-frequency range of 30–80 Hz and a 6-Hz band from the high-gamma-frequency range of 80–150 Hz. In all cases this represented <10% of the frequency range contributing to those bands.

#### Data analysis.

All data analysis was performed offline using custom MATLAB (Mathworks) scripts. During slow oscillations, high-frequency oscillations occur transiently during the Up-state and are absent during the Down-state. Thus to investigate these nested oscillations in more detail it was first necessary to detect the UDS.

#### UDS detection.

**Fig. 1.**
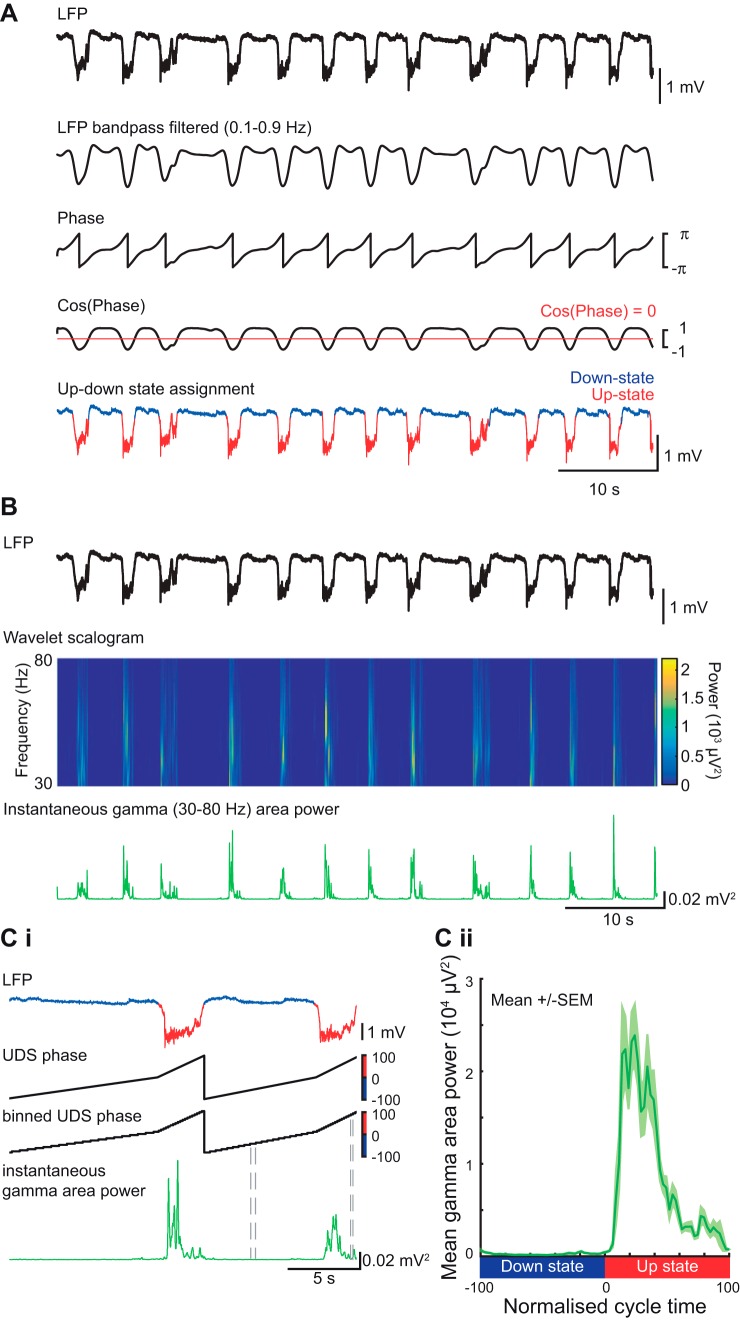
Up-Down state detection and alignment of fast oscillation power. *A*: illustration of Up- Down-state detection method. The local field potential (LFP) was filtered for the slow oscillation band. A threshold was set on the cosine of the slow oscillation phase to distinguish between Up- and Down-states leading to Up-and Down-state (UDS) assignment in the LFP trace. *B*: LFP trace (black) and wavelet time-frequency representation of the same signal (only gamma-frequency band is shown). The instantaneous gamma-frequency area power (green trace) was calculated from the wavelet scalogram. *Ci*: illustration of alignment method used for averaging gamma power over several cycles (despite variability in UDS cycle length). To align the instantaneous gamma power to the normalized UDS cycle, a UDS phase vector was calculated after the UDS detection. This vector was then binned into 40 phase bins per state and was used to calculate gamma power for each normalized cycle. *Cii*: gamma-frequency area power aligned to the normalized UDS cycle (mean over all cycles from 1 animal).

UDS detection was performed using the phase of the slow oscillation, as described previously (Massi et al. 2012), except that the Hilbert transform (rather than the wavelet transform) was used to calculate the phase of the slow oscillation (Fig. 1*A*). The LFP was first band-pass filtered (0.1–0.9 Hz) and the instantaneous phase ϕ(*t*) was calculated using the Hilbert transform. The threshold to discriminate between the Up-state and Down-state was cos[ϕ(*t*) = 0]. To qualify as an Up-state the duration of the event had to exceed 300 ms and the average amplitude of the Up-state over all channels was required to be larger than 0.5 mV. To assess the oscillatory power with respect to the UDS, where the UDS cycles had different lengths, the instantaneous power was aligned to a “normalized” Down-state to Up-state cycle, as described below.

#### Calculation of the UDS phase vector.

Time points of state transitions were calculated from the UDS detection logical vector. A cycle always consisted of a Down-state and an Up-state and contained three transitions: *transition 1*: Up-to-Down transition (i.e., end of the Up-state to beginning of the downstate); *transition 2*: Down-to-Up transition (i.e., end of the Down-state to beginning of the Up-state); and *transition 3*: Up-to-Down transition (i.e., end of the Up-state to beginning of the downstate). A phase vector was calculated that assigned −100 to the time point of *transition 1*, 0 to the time point of *transition 2*, and +100 to the time point of *transition 3*. The time points in between were filled with linearly spaced intermediate values, so that a phase vector was achieved with linear phase progression during the Down-state and linear phase progression during the Up-state. All UDS were then divided into 40 bins per state (Fig. 1*Ci*), and these were used to align other parameters such as fast oscillation power and slow oscillation amplitude.

#### Calculation of power at higher frequencies.

Oscillations in the delta (2–4 Hz)-, spindle (6–15 Hz)-, beta (20–30 Hz)-, gamma (30–80 Hz)-, and high-gamma (80–150 Hz)-frequency bands were calculated using a continuous wavelet transform on the LFP data (Fig. 1*B*), using a complex Morlet wavelet (Massi et al. 2012). The instantaneous area power was then calculated using trapezoidal numerical integration over all frequencies in the given band. The mean power per bin of the UDS phase vector was calculated for each UDS cycle, leading to the alignment of the power to a normalized cycle (Fig. 1*C*). This compensated for the variable lengths of UDS cycles within a particular data segment and thus allowed a mean power and standard error over the normalized UDS cycle to be calculated for each electrode channel in a particular animal. The latency to peak power was calculated as the time difference between Up-state onset and peak power in a specific band. For calculating this latency, the instantaneous power was smoothed (moving average filter, 0.2-s windows). These latency profiles were obtained using nonnormalized Up-states, using the maximal peak within the first 1.5 s after Up-state onset.

#### Calculation of slow oscillation amplitude.

The amplitude of the slow oscillation was calculated by filtering the LFP (0.1-0.9 Hz) and aligning the trace to the normalized UDS cycle. The mean (over all cycles) of this UDS was calculated, and the slow oscillation amplitude was calculated as the peak-to-trough amplitude (maximum during Up state − minimum during Down state) of this average waveform.

#### Cross correlation and cross spectral phase.

The cross correlation between two time series was calculated using MATLAB function “xcorr.m” and then normalized. The time lag between two signals was calculated as the time lag at the peak of the cross correlation. The cross power spectral density of the two signals was calculated using the MATLAB function “cpsd” with the following parameters: 30-s Hamming window, 50% overlap, and FFT length of 16,384. The phase angle was then calculated and plotted vs. frequency.

#### Data grouping.

All parameters were averaged from a continuous 120-s recording epoch within an animal yielding a mean value for each animal and each recording site. Statistical analysis was then performed on these mean values. In view of the small sample sizes normality could not be reliably assessed; therefore, nonparametric statistical methods were used. Group data are presented using box plots, in which the median is plotted as a line, the box constitutes the interquartile range (25–75 IQR), and the whiskers represented the most extreme values.

A Wilcoxon signed rank test was used to compare two related samples with respect to one factor. Friedman’s test (one-way nonparametric ANOVA) was used to compare more than two related samples with respect to one factor and reported as χ^2^ with degrees of freedom (df). The Tukey’s multiple comparisons test was used for post hoc comparisons. The Wilcoxon signed rank test and Friedman’s test were performed in MATLAB (MathWorks). Data are reported as significantly different with *P* < 0.05, *P* < 0.01, and *P* < 0.001.

## RESULTS

### 

#### Slow oscillations in different mPFC subregions.

**Fig. 2.**
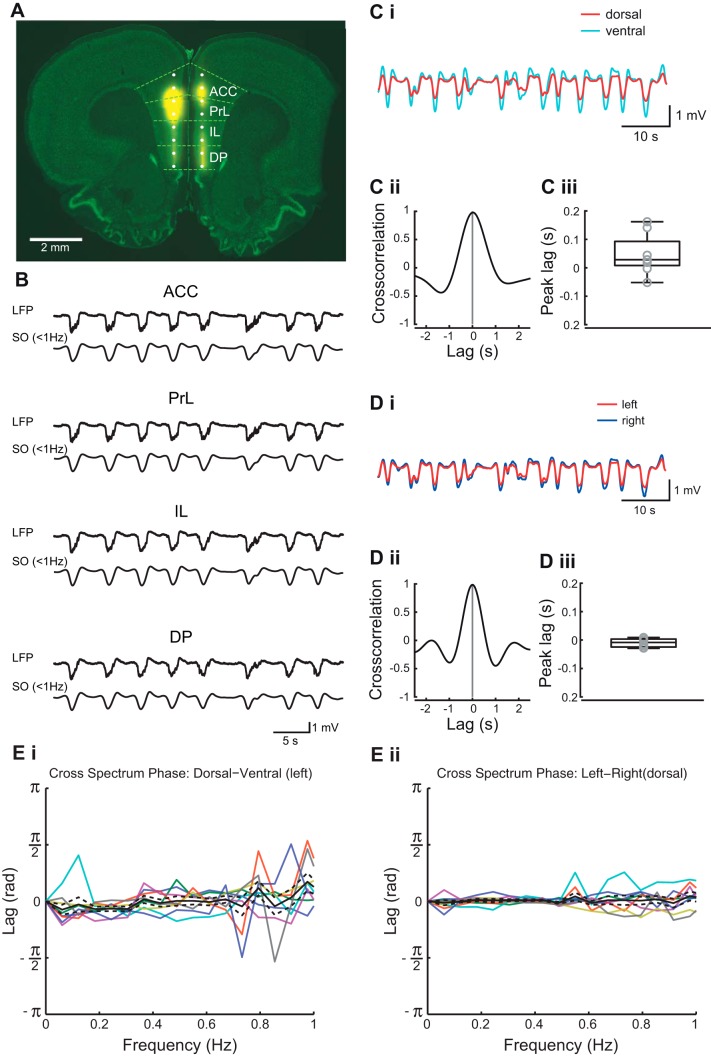
Recording sites and slow oscillation properties in medial prefrontal cortex (mPFC). *A*: coronal section through the rat mPFC indicating the position of the recording sites of the 16-channel dual shank silicon probe. Yellow represents DiI labeling of the electrode track. The background stain is green fluorescent Nissl. The light green dotted lines indicate the borders of the mPFC subregions anterior cingulate cortex (ACC), prelimbic cortex (PrL), infralimbic cortex (IL), and dorsal peduncular cortex (DP). The right hemisphere was lesioned before cutting for the purpose of tracking the hemisphere. *B*: examples of 60-s recordings of LFP and filtered traces of slow oscillations (SO) recorded simultaneously in each of the 4 mPFC subregions. *C*: example of 40 s LFP traces (*i*) from most dorsal (ACC) and most ventral (DP) recording sites, filtered for the slow oscillation; cross correlation of the 2 traces (*ii*) and box plot (*iii*) showing peak lags of cross correlation. *D*: example of 60 s LFP traces (*i*) from most dorsal (ACC) sites in the left and right hemisphere, filtered for the slow oscillation; cross correlation of the 2 traces (*ii*) and box plot (*iii*) showing peak lags of cross correlation. *E*: cross spectrum phase analysis between the most dorsal (ACC) and most ventral (DP) recording sites (*i*) and between the most dorsal (ACC) sites (*ii*) in the right and left hemisphere. Solid black lines and dashed lines represent means ± SE, respectively, with individual data points from each experiment in colored lines.

Bilateral field potential recordings were made from two sites in each subregion of the mPFC: ACC, PrL, IL, and DP in both hemispheres (see methods). Post hoc histology confirmed that the shanks were equidistant from the midline so that the laminar position of the shank was in layer III of the mPFC in both hemispheres (Fig. 2*A*). Up-states correspond to intracellular depolarization and periods of maximal neuronal firing (Steriade et al. 1996a,b; Sanchez-Vives and McCormick 2000; Hughes et al. 2002). In extracellular field recording the equivalent event is seen as a negative deflection in the LFP, associated with nested fast oscillations that we will refer to as an Up-state (Fig. 2*B*). Down-states reflect intracellular hyperpolarization and periods of less intense neuronal firing (Steriade et al. 1996a,b; Sanchez-Vives and McCormick 2000; Hughes et al. 2002). In extracellular field recording the Down-state is observed as a positive deflection, associated with little nested fast oscillatory activity (Fig. 2*B*). Although we have not recorded intracellularly in this study, we will refer to these equivalent phasic changes in the slow oscillations as Up- and Down-states. Under urethane anesthesia cortical activity exhibits changes between a rapid eye movement (REM)-like sleep state and a slow wave activity (SWA) deep sleep-like state, in both rats (Clement et al. 2008) and mice (Pagliardini et al. 2013a,b). We found that at the dose used (see methods), urethane evoked long-lasting (~1.5 - 2 h) periods of slow oscillations without spontaneous state changes.

We first assessed the dorsal-to-ventral synchrony of the slow oscillation in the mPFC and revealed that Up-states occurred synchronously within the mPFC (Fig. 2*C*). UDS transitions were determined as described in methods. Up-states occurred simultaneously in each subregion (Fig. 2, *B* and *C*), and this was confirmed by cross correlation calculated between the slow oscillations in ACC and DP (Fig. 2, *Cii* and *Ciii*), which showed no consistent dorsal-to-ventral time lag (*P* > 0.05 Wilcoxon signed-rank test, median: 19 ms, IQR: −0.5 – 80.5, *n* = 8). Slow oscillations were also found to be highly synchronous across the two hemispheres (Fig. 2*D*). The interhemispheric cross correlation for electrodes in ACC (Fig. 2, *Dii* and *Diii*) was not significantly different from zero (*P* > 0.05, Wilcoxon signed rank test, median: −0.5 ms, IQR: −24.5 – 3.5, *n* = 8). In addition, the cross spectrum phase analysis showed a phase difference close to zero between both the dorsal and ventral (Fig. 2*Ei*) recording sites and between the right and left hemisphere (Fig. 2*Eii*).

#### Up-Down state parameters in mPFC.

**Fig. 3.**
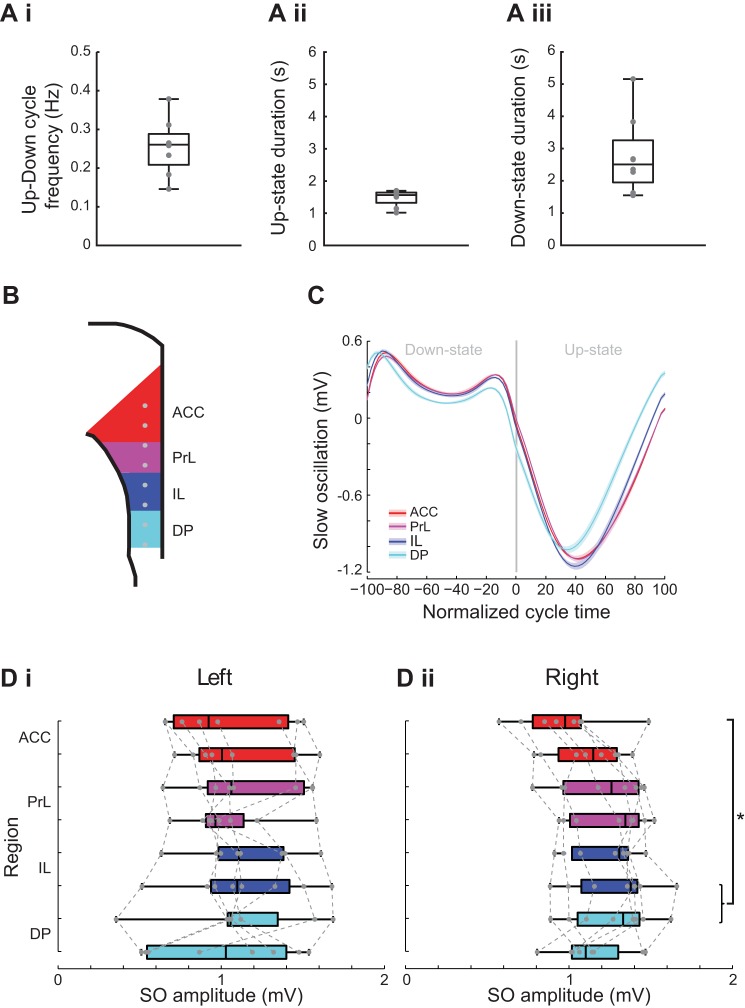
Subregional comparison of slow oscillation amplitude. *A*: box plots showing Up-Down cycle frequency (*i*), Up-state duration (*ii*), and Down-state duration (*iii*). *B*: schema of the mPFC with indication of recording sites (gray dots) within each subregion color coded (ACC, red; PrL, purple; IL, dark blue; DP, light blue). *C*: example of the slow oscillation in each region aligned to the normalized Down-state-Up-state cycle. Solid line shows means ± SE (shaded region) over all cycles in the analyzed data segment from 1 animal (regions color coded as indicated in the above schema). *Di*: box plot showing slow oscillation peak-to-peak amplitude in the left hemisphere in each subregion of the mPFC. *Dii*: box plot showing slow oscillation peak-to-peak amplitude in the right hemisphere in each subregion of the mPFC.

As the slow oscillations were synchronous across all mPFC regions data from just one channel in one region (ACC) was used to assess the frequency properties of the Up-Down states including, Up-Down cycle frequency, Up-state duration, and Down-state duration (Fig. 3*A*). The frequency of the slow oscillation (Fig. 3*Ai*) was highly consistent between animals (median frequency 0.26 IQR: 0.21 − 0.29 Hz, *n* = 8). The Up-state duration was significantly shorter than the Down-state duration (Fig. 3, *Aii* and *Aiii*): median Up-state duration of 1.56 s (IQR: 1.32 − 1.64 s, *n* = 8) vs. a Down-state duration of 2.51 s (IQR: 1.94 − 3.26 s, *n* = 8, *P* < 0.05, Wilcoxon signed rank test).

To examine whether the amplitude of the slow oscillation was consistent across mPFC regions we compared the amplitude recorded from two sites in each region (Fig. 3*B*), measured from peak-to-trough, aligned to the normalized UDS cycle (see methods) (Fig. 3*C*). The absolute amplitude of the slow oscillation varied between experiments but data for the left hemisphere showed there was no consistent amplitude difference across any subregion of the mPFC [χ^2^(7) = 6.25, Friedman’s test, *P* > 0.05, *n* = 8, Fig. 3*Di*]. Data for the right hemisphere also varied between experiments, and in this data set there was a small, but significant, difference between dACC and dIL/vDP [χ^2^(7) = 18.33, Friedman’s test, *P* < 0.05, *n* = 8, Fig. 3*Dii*].

#### Nested Up-state oscillations.

**Fig. 4.**
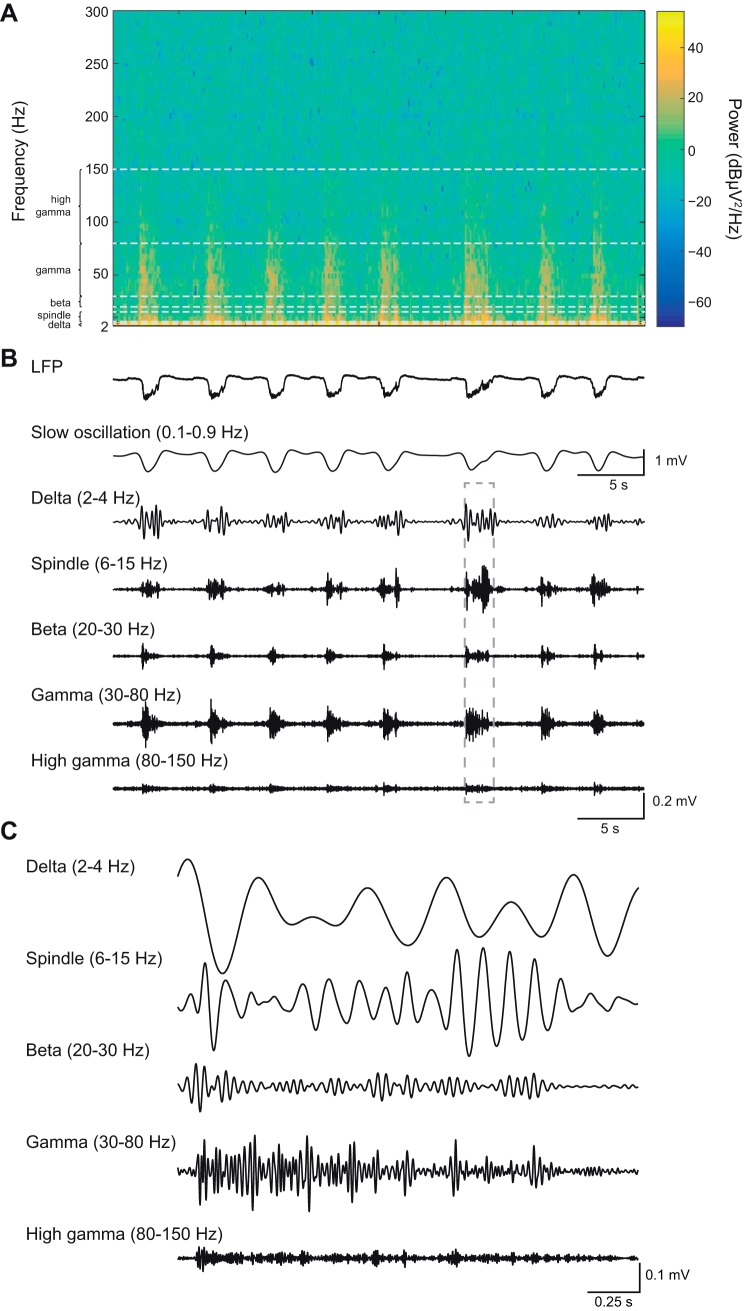
High-frequency activity occurred nested on the Up-state. Example spectrogram shows the time-frequency representation of a 40-s LFP segment recorded in ACC (*A*) and the corresponding (line-noise filtered) LFP trace (*B*). The low- and high-frequency components of the LFP are shown by filtering for specific frequency bands. Note that the high-frequency oscillations occur during the negative phase of the slow oscillation deflection (i.e., the Up-state). *C*: magnification of selected sections (indicated by gray dotted box in *B* of the filtered LFP.

Next, we examined the fast oscillatory activity nested on the Up-state (Steriade et al. 1996a,b; Ruiz-Mejias et al. 2011; Valencia et al. 2013). Oscillatory activity in the delta (2–4 Hz)-, spindle (6–15 Hz)-, beta (20–30 Hz)-, gamma (30–80 Hz)-, high-gamma (80–150 Hz)-frequency bands (Fig. 4, *A*–*C*) present on the Up-state was compared between all subregions of the mPFC. For clarity, we predominantly describe data only from the left hemisphere recordings. However, the data for the right hemisphere recordings are shown in each figure for comparison, and their statistics are summarized in Table 1.

**Table 1.** Statistical data for right hemisphere power and latency values in each frequency band

Right Hemisphere	χ^2^	*P*
Delta power	14.46	<0.05
Delta latency	8.65	>0.05
Spindle power	29.12	<0.001
Spindle latency	18.41	=0.01
Beta power	46.21	<0.001
Beta latency	23.44	<0.01
Gamma power	52.58	<0.001
Gamma latency	27.26	<0.001
High gamma Power	47.83	<0.001

#### Subregional profile of Up-state delta power and latency in mPFC.

**Fig. 5.**
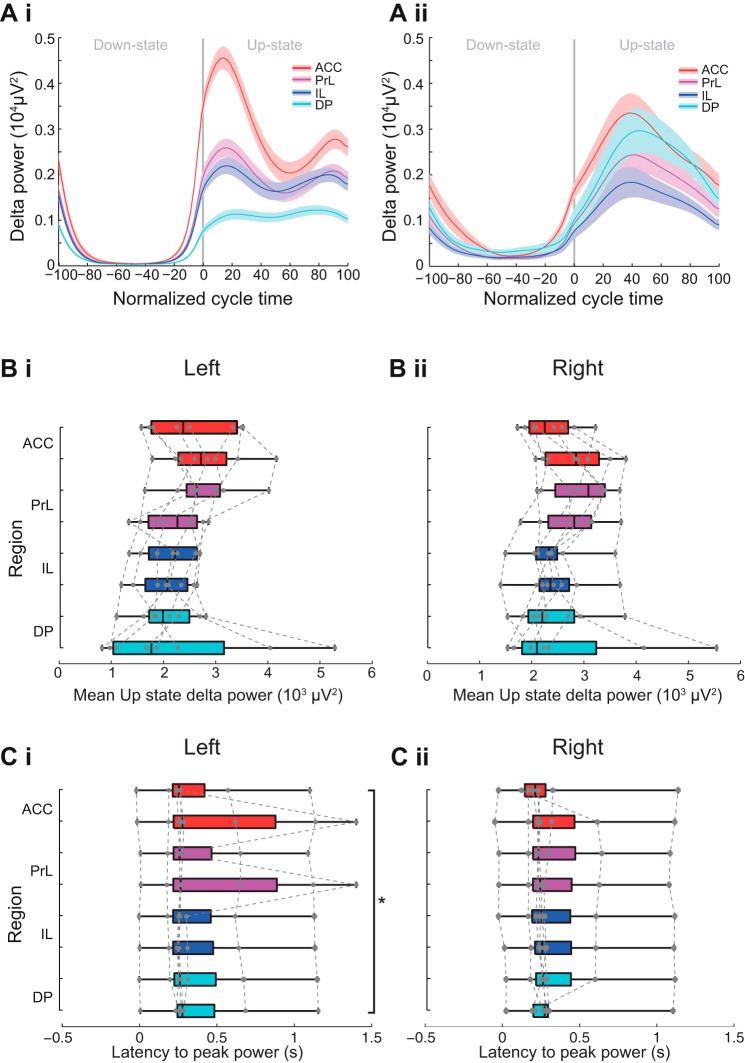
Subregional profile of Up-state delta-frequency oscillation power and latency. *Ai* and *Aii*: 2 different examples of delta power across the normalized slow oscillation cycle. Solid line shows means ± SE (shaded region) over all cycles in the analyzed data segment from 2 different animals (regions color coded as indicated in the schema in Fig. 3*B*). Boxplot showing subregional profile of mean Up-state spindle-frequency power in the left (*Bi*) and right (*Bii*) hemisphere (gray dots show individual data points). Boxplot showing subregional profile of peak Up-state delta-power latency in the left (*Ci*) and right (*Cii*) hemisphere (gray dots show individual data points).

The power of the oscillations in the delta range did not show any consistent subregional pattern (Fig. 5 and see Fig. 10). Although statistical analysis revealed that there was a significant subregional difference in mean Up-state delta-frequency power [χ^2^(7) = 16.17, *P* < 0.05, Friedman’s test, *n* = 8], post hoc analysis did not reveal significant differences and the variation was smaller than for the other frequency bands. The latency to the peak delta power varied by mPFC subregion [χ^2^ (7) = 14.71, *P* < 0.05, Friedman’s test, *n* = 8]. Post hoc tests showed that latency to peak delta power was significantly shorter in ACC than DP but only in the left hemisphere (Tukey test, *P* < 0.05).

#### Subregional profile of Up-state spindle power and latency in mPFC.

**Fig. 6.**
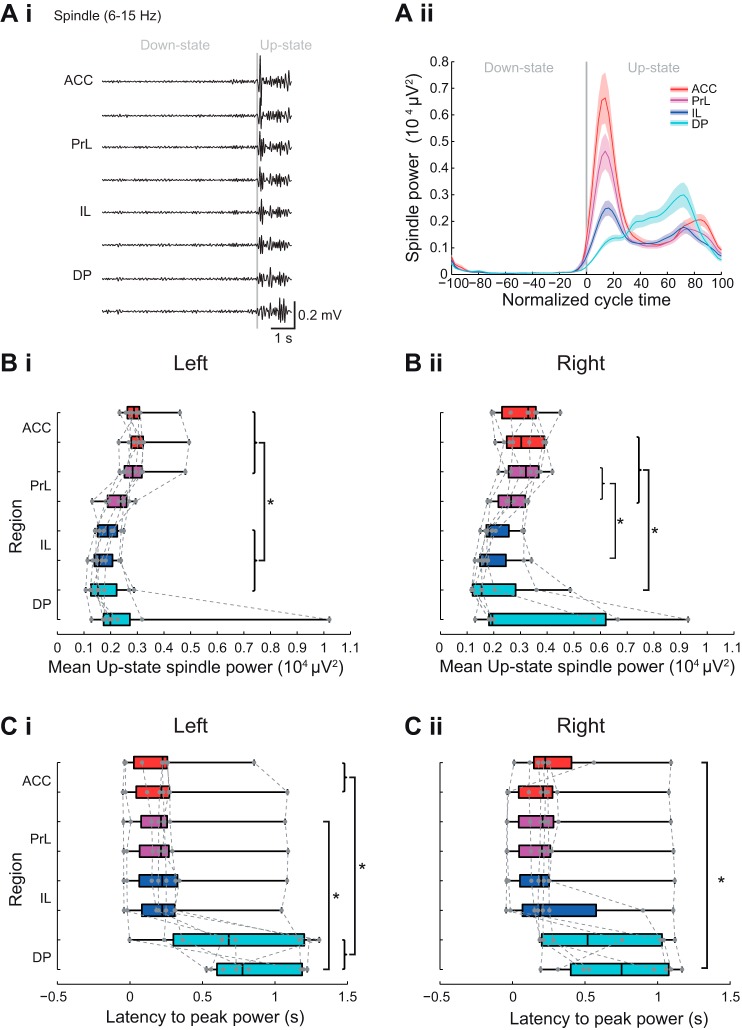
Subregional profile of Up-state spindle-frequency oscillation power and latency. *Ai*: example of filtered LFP traces showing spindle-frequency oscillations over 1 cycle of Down- to Up-state. *Aii*: example of spindle power across the normalized slow oscillation cycle. Solid line shows means ± SE (shaded region) over all cycles in the analyzed data segment from 1 animal (regions color coded as indicated in the schema in Fig. 3*B*). Boxplots showing subregional profile of mean Up-state spindle-frequency power in the left (*Bi*) and right hemispheres (*Bii*) (gray dots show individual data points). Boxplots showing subregional profile of peak Up-state spindle-power latency in the left (*Ci*) and right (*Cii*) hemisphere (gray dots show individual data points).

The power of the oscillations in the spindle-frequency (6–15 Hz) range varied as a function of mPFC subregion with the highest power oscillations in ACC and PrL (Fig. 6, *A* and *B* and see Fig. 10). Statistical analysis confirmed a significant subregional difference in mean Up-state spindle-frequency power [χ^2^(7) = 37.62, *P* < 0.001, Friedman’s test, *n* = 8]. Post hoc tests showed that spindle power in the left dACC, vACC and dPrL was significantly greater (Fig. 6*Bi*) than in the dIL, vIL, and dDP (Tukey test, *P* < 0.05). This profile of dorsal-to-ventral area power changes occurred in all animals, and both hemispheres.

The most notable difference observed with oscillations in the spindle-frequency range was a change in the temporal profile of spindle power between mPFC subregions. Thus in ACC, PrL, and IL, the latency to peak spindle power showed a clear peak early after the onset of the Up-state (Fig. 6, *A* and *C*). In contrast, in DP the latency to peak spindle power was more variable, and spindle-frequency oscillations occurred predominantly in the latter half of the Up-state (Fig. 6, *A* and *C*). Statistical analysis for the left hemisphere (Fig. 6*Ci*) showed that the latency to the peak spindle power varied by mPFC subregion [χ^2^ (7) = 33.34, *P* < 0.001, Friedman’s test, *n* = 8]. Post hoc tests showed that latency to peak spindle power in ACC differed from DP and the latencies in the dPrL differed from the latencies observed in vDP (Tukey test, *P* < 0.05). Similar dorsal-to-ventral differences were seen in the right hemisphere (Fig. 6*Cii* and Table 1).

#### Subregional profile of Up-state beta power and latency in mPFC.

**Fig. 7.**
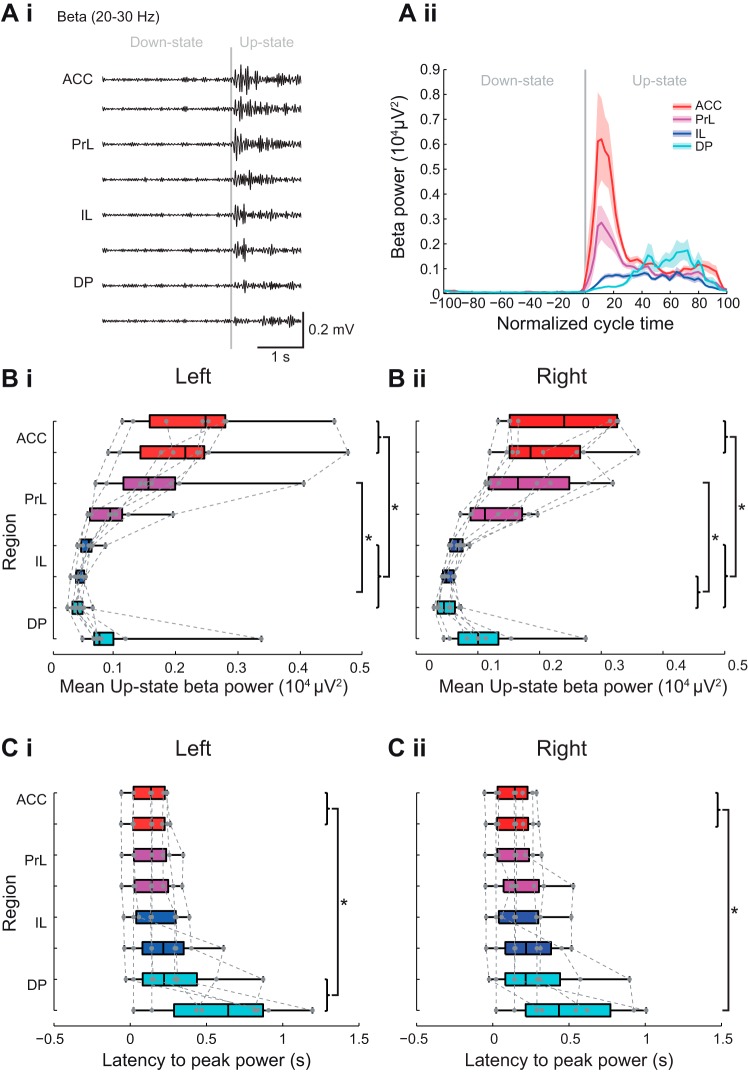
Subregional profile of Up-state beta-frequency oscillation power and latency. *Ai*: example of filtered LFP traces showing beta-frequency oscillations over 1 cycle of Down to Up-state. *Aii*: example of beta-frequency power across the normalized slow oscillation cycle. Solid line shows means ± SE (shaded region) over all cycles in the analyzed data segment from 1 animal (regions color coded as indicated in the schema in Fig. 3*B*). Boxplots showing subregional profile of mean Up-state beta power in the left (*Bi*) and right (*Bii*) hemisphere (gray dots show individual data points). Boxplots showing subregional profile of peak Up-state beta-power latency in the left (*Ci*) and right (*Cii*) hemisphere (gray dots show individual data points).

When we compared beta-frequency activity in each subregion of the mPFC we found that, as seen with spindle power outlined above, the largest beta power also occurred in the ACC and PrL (Fig. 7 and see Fig. 10). Statistical analysis of beta-frequency power (Fig. 7*Bi*) showed a significant subregional difference in the left hemisphere [*χ^2^*(7) = *48.96*, *P* < 0.001, Friedman’s test, *n* = 8]. Post hoc tests showed beta-frequency power in the ACC and dPrL was significantly higher than in vIL and dDP. The beta power in the ACC was also significantly higher than in the dIL (*P* < 0.05 Tukey test). A similar profile was seen in the right hemisphere (Fig. 7*Bii* and Table 1).

The temporal profile of beta power also varied across mPFC regions (Fig. 7*C*). In the dorsal mPFC regions, beta power occurred early and in a narrow time window after Up-state onset. However, in the vIL and DP regions, beta activity mainly occurred later in the Up-state, and the peak of beta power was less defined (χ^2^ = 27.91, *P* < 0.001, Friedman’s test, *n* = 8). Post hoc tests showed that the latency to peak power was significantly shorter in ACC than DP (Tukey test *P* < 0.05), with the same profile seen in the right hemisphere (Fig. 7*Cii* and Table 1).

#### Subregional profile of Up-state gamma power and latency in mPFC.

**Fig. 8.**
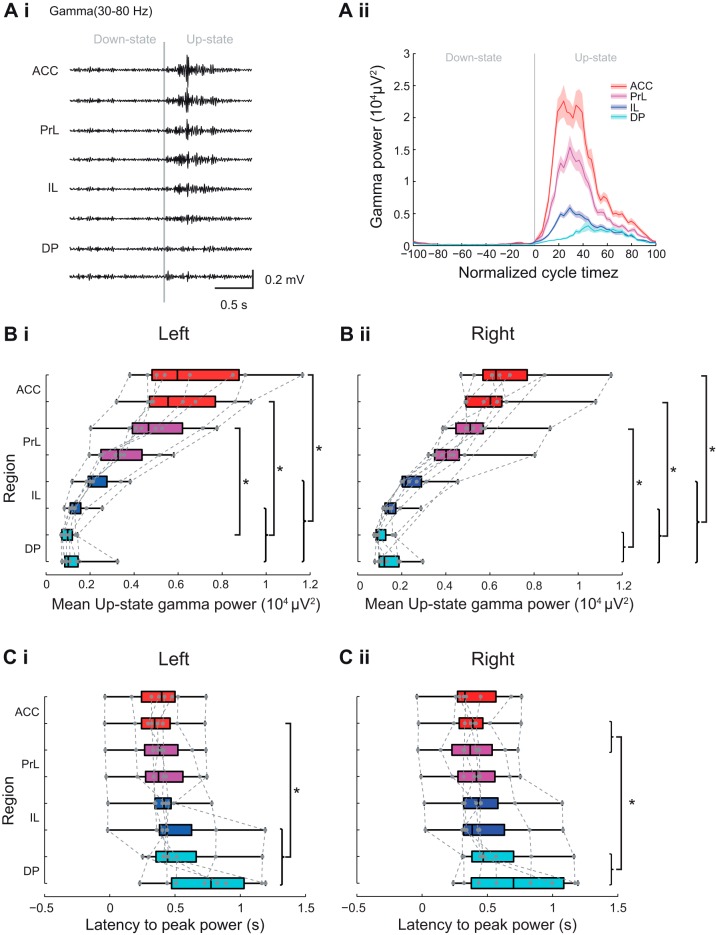
Subregional profile of Up-state gamma-frequency oscillation power and latency. *Ai*: example of filtered LFP traces showing gamma-frequency oscillations over 1 cycle of Down to Up-state. *Aii*: example of gamma-frequency power across the normalized slow oscillation cycle. Solid line shows means ± SE (shaded region) over all cycles in the analyzed data segment from 1 animal (regions color coded as indicated in the schema in Fig. 3*B*). Boxplots showing subregional profile of mean Up-state gamma power in the left (*Bi*) and right (*Bii*) hemisphere (gray dots show individual data points). Boxplots showing subregional profile of peak Up-state gamma-power latency in the left (*Ci*) and right (*Cii*) hemisphere (gray dots show individual data points).

The profile of gamma-frequency power changes across mPFC regions was similar to that described above for beta activity, with a much greater gamma power evident in the ACC and PrL regions, compared with the DP region (Fig. 8 and see Fig. 10). However, in contrast to the beta-frequency activity described above, gamma-frequency activity occurred over a greater portion of the Up-state (Fig. 8*A*). Statistical analysis of the mean Up-state gamma-frequency power (Fig. 8*Bi*) showed a significant subregional difference in power [χ^2^(7) = 52.88, *P* < 0.001, Friedman’s test, *n* = 8]. Post hoc analysis of the left hemisphere recording revealed that ACC differed from vIL and DP, dACC also differed from dIL, and dPrL differed from the dDP (Tukey test, *P* < 0.05). A similar pattern of dorsal-to-ventral differences was also seen in the right hemisphere (Fig. 8*Bii* and Table 1).

In contrast to the beta-frequency activity described above, gamma-frequency activity was less tightly locked to the Up-state onset and occurred over a large portion of the Up-state (Fig. 8*A*). In ventral mPFC regions, the peak of gamma power was less well defined, particularly in DP. The latency to the peak gamma-frequency power varied (Fig. 8*Ci*) significantly with subregion [χ^2^ (7) = 32.42, *P* < 0.01, Friedman’s test, *n* = 8]. Post hoc tests showed that the latency to peak power was significantly longer in the vIL, dDP, and vDP than in the vACC (Tukey test, *P* < 0.05) with, again, a similar pattern observed in the right hemisphere (Fig. 8*Cii* and Table 1).

#### Subregional profile of Up-state high-gamma power in mPFC

**Fig. 9.**
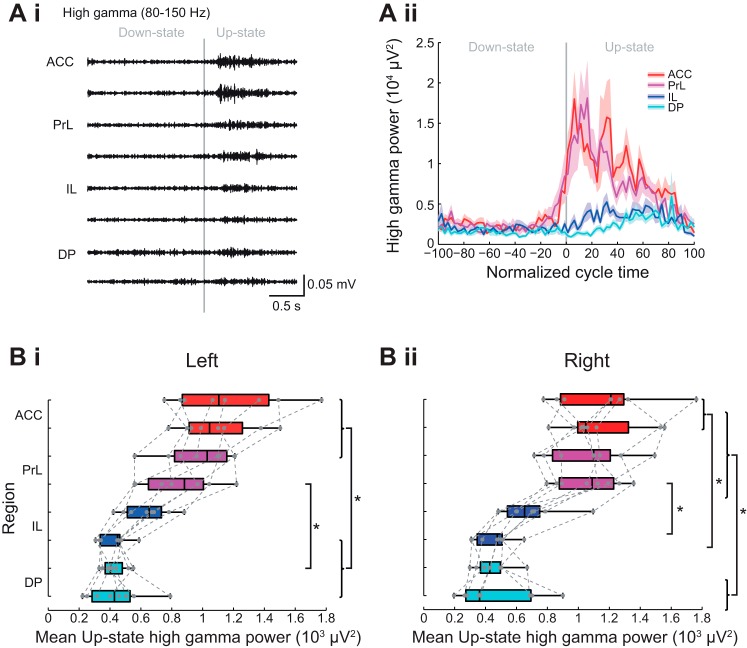
Subregional profile of Up-state high-gamma-frequency oscillation power. *Ai*: Example of filtered LFP traces showing high frequency oscillations over 1 cycle of Down to Up-state. *Aii*: example of high-gamma-frequency power across the normalized slow oscillation cycle. Solid line shows means ± SE (shaded region) over all cycles in the analyzed data segment from 1 animal (regions color coded as indicated in the schema in Fig. 3*B*). Boxplots showing subregional profile of mean Up-state high-gamma power in the left (*Bi*) and right (*Bii*) hemisphere (gray dots show individual data points). Boxplots showing subregional profile of peak Up-state high-gamma-power latency in the left (*Ci*) and right (*Cii*) hemisphere (gray dots show individual data points).

**Fig. 10.**
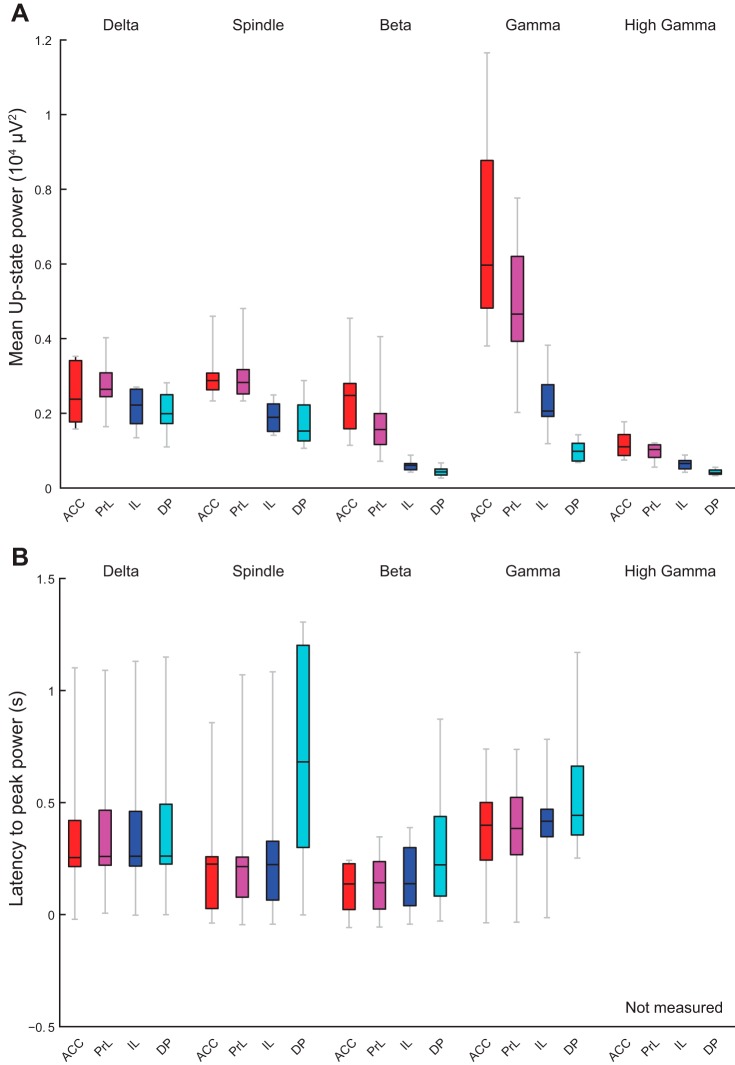
Subregional comparisons of power and latency in each frequency band and latency to peak power. Boxplots replot the median area power (*A*) and latency (*B*) values in each frequency band for 1 recording site as shown in Figs. 5–9 for direct comparison across regions. Regions color coded as indicated in the schema in Fig. 3*B*.

High-gamma-frequency activity was present in all regions but the power was largest in ACC and PrL (Fig. 9 and see Fig. 10). In contrast to the other fast oscillations described above, in the dorsal regions high-gamma-frequency activity was seen over a large proportion of the Up-state (Fig. 9*A*). Group data confirmed a statistically significant subregional difference (Fig. 9*Bi*) in Up-state high-gamma-power in the left hemisphere (χ^2^ (7) = 47.25, *P* < 0.01, Friedman’s test). Post hoc tests showed that ACC and dPrL differed from vIL and DP and vPrL differed from dDP (Tukey test, *P* < 0.05). Overall, similar dorsal vs. ventral area power differences were seen in the right hemisphere (Fig. 8*Bii* and Table 1).

As can be seen in Fig. 8*A* the peak power of high-gamma activity was poorly defined in all regions, although in ACC and PrL the majority of the activity occurred early on the Up-state, whereas in the IL and DP activity was quite widely distributed over the Up-state (Fig. 9*A*). However, because of the lack of any clear peak, statistical analysis on the latency to peak power was not performed for high-gamma-frequency activity.

#### Overview of fast Up-state-associated oscillations.

In Fig. 10 we have replotted the same median values of area power and latency (for one recording site per region) from the left hemisphere, as shown in Figs. 5–9 above, to allow direct comparison of different frequency bands. With the exception of the delta-frequency activity, all other oscillations were largest in the most dorsal regions. When comparing the latency to peak power (Fig. 10*B*), activity at beta frequency occurred earliest on the Up-state in all regions, followed by spindle, delta, and finally gamma activity. There were no consistent variations in the latency of the different oscillations, with the notable of exception of spindle activity, which occurred much later on the Up-state in the DP than other, more dorsal regions of mPFC.

## DISCUSSION

In the present study we examined the potential differences between the mPFC subregions with respect to the power and timing of the oscillatory activity nested on the Up-state of the slow oscillations recorded under urethane anesthesia. We found that slow oscillations of similar amplitude were present in all subregions and were highly synchronous throughout the dorsal-to-ventral extent of the mPFC, as well as across hemispheres. Interestingly, with the exception of the delta-frequency activity, the faster oscillations on the Up-state showed marked subregional differences in both their power and temporal profile. Of particular note, we found the power of the high-frequency activity to be greatest in the dorsal ACC and PrL regions of the mPFC with much less activity in ventral regions, especially DP. Moreover, the temporal distributions of the spindle-, beta-, and gamma-frequency oscillations during the Up-state were also very different: in the dorsal regions the peak power had a short latency in contrast to the more ventral regions where peak power occurred later during the Up-state.

### 

#### Subregional properties of slow oscillations in the mPFC.

Although the slow oscillation can persist in the absence of thalamic input, synchronized activity requires an intact thalamo-cortical circuit (David et al. 2013; Lemieux et al. 2014). In this study we found that the slow oscillation recorded under urethane anesthesia is highly synchronous throughout the dorsal-to-ventral extent of the mPFC. This is consistent with findings from other cortical regions that show precise synchronization of slow oscillations across the dorsal surface of the neocortex (Massimini et al. 2004; Volgushev et al. 2006; Ruiz-Mejias et al. 2011; Sheroziya and Timofeev 2014). Thus, despite the fact that in the mPFC the anatomical arrangement of the cortex is perpendicular to that across the dorsal surface, slow oscillation synchrony is preserved between mPFC regions. In support of our data Kuramoto et al. (2016) have recently shown that a single neuron in the thalamic mediodorsal (MD) nucleus has projections that can arborize in multiple subregions of the mPFC, which would enable activation of neuronal ensembles to be synchronized across subregions.

In our recordings, slow oscillations were also highly synchronous between the two hemispheres of the mPFC. Mohajerani et al. (2010) reported a similar phenomenon in mouse neocortex in both the quiet-wake state and under urethane anesthesia and demonstrated that cortical interhemisphere synchrony was greatly reduced, although not absent, in a genetic mouse model lacking callosal connections. These authors proposed that the residual synchrony in the acallosal mice reflected a synchronizing role for other subcortical inputs, perhaps including thalamus. With respect to our data, the slow oscillation synchrony we observed likely reflects both callosal connections and bilateral projections from the MD nucleus of the thalamus to layer III of mPFC (see Vertes et al. 2015 for review), as well as a high degree of interconnectivity between subregions (Heidbreder and Groenewegen 2003).

#### Subregional differences in Up-state-associated oscillations.

Several studies have now demonstrated that during the Up-state different frequencies of oscillation are evident in the local field potential, particularly in the delta-, spindle-, beta-, gamma-, and high-gamma-frequency activity (Steriade et al. 1996a,b; Hartwich et al. 2009 Ruiz-Mejias et al. 2011; Valencia et al. 2013; Gardner et al. 2013). Here, we found significant dorsal-to-ventral differences in the power of these nested fast oscillations. We observed these differences in both the left and right hemisphere recordings made with a two shank electrode with fixed spacing, both shanks of which were histologically confirmed to be in layer III. The fact that we found subregional differences, in the absence of systematic differences in the power of the slow oscillations, suggests that aspects of these nested oscillations are independent of the slow oscillations.

Delta-frequency activity, which predominates in slow wave sleep and under anesthesia, can be generated by a well understood interplay of ionic currents in thalamocortical cells (Amzica et al. 1992; Hughes et al. 2002). Interestingly, we noted that delta power began to increase just before the Up-state onset and persisted for a short period after the transition to Down-state. This could possibly reflect a thalamic delta source, which might actually be the trigger for the transition to an Up-state. However, there is also clear evidence for an independent cortically generated delta rhythm (Vassalli and Dijk 2009; Carracedo et al. 2013). In the rat mPFC delta oscillations recorded under urethane anesthesia were found to persist following thalamic inactivation (Kiss et al. 2011). Interestingly, in humans it has been shown that closely interacting regions (entorhinal cortex and hippocampus) have their own separate delta-oscillation generators (Mormann et al. 2008). Our results showed that mPFC subregions varied quite widely in the magnitude of delta power they displayed and that no single subregion consistently showed the highest magnitude of delta power, possibly reflecting different mechanistic origins of the delta activity in different experiments.

Spindle-frequency activity arises from the thalamus through a rhythmic interaction between thalamocortical neurons and GABAergic inhibitory interneurons in the thalamic reticular nucleus (for review see McCormick et al. 2015). One recent study in somatosensory cortex showed that the likelihood of spindle occurrence has a bimodal distribution, with a higher probability at both the beginning and the end of the Up-state (Valencia et al. 2013). In addition, pyramidal cells that fire early or late during a spindle oscillation in the mPFC have been reported (Gardner et al. 2013), suggesting that distinct networks may be involved in spindle generation. Here we found that in the dorsal mPFC, where spindle-frequency oscillations were large, the latency to peak power occurred early during the Up-state with little variability, although in some cases a second peak in spindle activity was seen later during the Up-state. In contrast, in the DP the latency to peak spindle power was highly variable, often occurring late during the Up-state. The temporal dissociation between the times of occurrence of spindles in the ACC vs. DP suggests possibly different types of spindle activity within the mPFC (Mölle et al. 2011).

Beta activity was also present in the mPFC and again the power was greatest in the ACC and lowest more ventrally. Interestingly, Ruiz-Mejias et al. (2011) reported beta power to be much greater in ACC in anesthetized mice compared with other (non-mPFC) cortical regions. Taken together these data suggest ACC may be unusual among cortical regions in having a high level of beta-frequency activity. Beta-frequency activity in the mPFC may reflect the role of this region in top-down “cognitive control” (Shenhav et al. 2013), since beta-frequency oscillations have been associated with long-range interactions (Kopell et al. 2010; Kopell et al. 2014). Our finding of lower beta activity in DP in vivo under anesthesia appears to conflict with our recent in vitro study where beta-frequency activity in DP was larger than that seen in PrL (Glykos et al. 2015). However, in that study oscillations were evoked by a combination of cholinergic receptor activation (using carbachol) and kainate receptor activation to model wake-state oscillations. During slow wave sleep or, as in our recordings under anesthesia, the levels of acetylcholine and other neuromodulators are different to those in the awake-state, which could lead to large differences in intrinsic ion channel properties, for example, affecting the relative power of different network oscillations. This raises the possibility there might be mechanistically different types of beta activity.

Gamma (30–80 Hz)-frequency activity is known to depend on the activity of fast-spiking interneuron populations (Whittington et al. 2011). The greater power of the gamma-frequency activity we observed in dorsal mPFC regions (ACC and PrL) could reflect a larger number of neurons activated and/or stronger neuronal connectivity, resulting in increased synchronization of activity in these regions, relative to more ventral parts. High-gamma-frequency activity was the least prominent of the high-frequency oscillations but, as with other frequencies, the power was greatest in the most dorsal subregions. One study demonstrated that most of the power in the 10- to 100-Hz range arises from synchronised inhibitory potentials during the Up-state (Hasenstaub et al. 2005) suggesting there may be more synchronized inhibition in dorsal regions. In addition, the larger power of both beta- and gamma-frequency activity in the dorsal ACC and PrL regions might reflect a dorsal origin to the generation of these frequencies of activity, which then propagates to more ventral regions. Differences in the power of the fast oscillations between subregions may also reflect the extent and nature of afferent inputs to each subregion. For example, the lateral MD nucleus has extensive arborizations to both ACC and PrL, while the medial MD projects to PrL and IL (Vertes et al. 2015; Kuramoto et al. 2016; Alcaraz et al. 2016), but we still know very little about inputs to DP.

The different, but consistent, latencies for peak spindle, beta, and gamma power we have observed in the dorsal mPFC regions suggest that different neurons may be involved in generating the different types of activity. Our data also demonstrated that, in the dorsal mPFC regions, peak beta power occurred during a narrower part of the Up-state cycle compared with the gamma activity and at a shorter latency than the gamma-frequency peak power. Beta- and gamma-frequency activity may depend on different interneuron populations, that give rise to inhibitory synaptic potentials, with different decay time constants (Roopun et al. 2008). The different profile of activation of beta and gamma activity on the Up-state observed here, therefore, suggests that potentially different interneuron and/or pyramidal cell populations may be activated in a distinct temporal order. Neurons recorded in both anesthetized and awake rats have been shown to exhibit a broad distribution of spike latencies after Up-state onset, with each neuron having a distinct spike pattern (Luczak et al. 2007). In a further study recorded only under anesthesia, both pyramidal cells and interneurons recorded at the same site in the neocortex had different latencies to first spike during the Up-state, and both cell classes could fire either early or late during the Up-state (Luczak and Barthó 2012). Two kinds of fast-spiking interneurons have been distinguished in cortical regions in vivo that either fire early or later during the Up-state (Puig et al. 2008). Different subtypes of GABAergic interneurons have also been shown to synchronise differently to spindle activity in the mPFC (Hartwich et al. 2013). Varying proportions of pyramidal and interneuronal subtypes, in the different mPFC regions, could therefore contribute to the different timings of peak oscillatory activity we observed. Overall our data support the notion that different cell assemblies are coactivated in a distinct order during an Up-state, which likely reflects both differences in the local neuronal network, as well as the different inputs and functions of mPFC subregions.

#### Limitations of urethane anesthesia.

Urethane is widely used to study sleep-related activity as several features of natural sleep, including spontaneous alternations in brain state (Pagliardini et al. 2013a,b), and spindle-frequency oscillations (Barthó et al. 2014) are reproduced under urethane anesthesia. Although in some brain regions urethane has been reported to affect GABA and glutamate function (McCardle and Gartside 2012), in the cortex urethane had little effect on these neurotransmitter systems and is thought to mediate its anesthetic actions by modulating intrinsic potassium conductances, thus depressing neuronal excitability (Sceniak and MacIver 2006). Although we believe urethane is unlikely to account for the subregional differences observed in this study, further experiments in unanesthetized animals are needed to confirm our findings are relevant in natural sleep.

#### Conclusions.

We demonstrate that the dynamics of Up-state associated fast oscillations show differences between dorsal and ventral mPFC. The higher power for all frequency bands in dorsal regions may be due to functional interactions between mPFC subregions in vivo through which dorsal regions may suppress the generation of fast oscillations in more ventral regions. Certainly there is evidence for strong interregional interactions in vitro (van Aerde et al. 2008), but further studies using inactivation of specific subregions would be required to reveal the extent, and direction, of interregional interactions within the mPFC in vivo. Alternatively, as outlined above, the dorsal to ventral differences may reflect different inputs to mPFC subregions (Vertes et al. 2015; Alcaraz et al. 2016). In addition, we have found that in the DP region the peak spindle, beta, and gamma power, relative to the onset of the Up-state, occurs much later and is more variable. Such marked differences in the power and timing of the fast oscillations suggest a unique role for network activity in the DP. Although little is known about the function of DP, which occupies a large area rostrocaudally (Paxinos and Watson 2007). consistent with our suggestion two recent detailed anatomical studies in rat and mouse suggest DP is distinct from other mPFC regions (Zingg et al. 2014; Akhter et al. 2014).

#### Future directions.

Abnormalities in sleep-related oscillations have been linked to a wide range of neurodegenerative conditions such as Alzheimer’s disease and Parkinson’s disease (Porter et al. 2015) as well as neuropsychiatric disorders including schizophrenia (Gardner et al. 2014; Manoach et al. 2015). In view of the key role mPFC dysfunction plays in these conditions, understanding the changes in sleep-related oscillations in the different subregions of the mPFC, could provide important insights into the pathological and functional changes associated with a range of diseases.

## GRANTS

S. Gretenkord was supported by a Wellcome Trust Ph.D. Studentship 092994/2/10/2.

## DISCLOSURES

No conflicts of interest, financial or otherwise, are declared by the author(s).

## AUTHOR CONTRIBUTIONS

S.G. performed experiments; S.G. analyzed data; S.G. prepared figures; S.G., A.R., M.A.W., S.E.G., and F.E.L. edited and revised manuscript; S.G., A.R., M.A.W., S.E.G., and F.E.L. approved final version of manuscript; A.R., M.A.W., S.E.G., S.G., and F.E.L. interpreted results of experiments; S.G. and F.E.L. drafted manuscript.
